# Gastric endometriosis associated with transverse colon endometriosis: a case report of a very rare event

**DOI:** 10.1007/s00404-014-3373-7

**Published:** 2014-07-17

**Authors:** V. Anaf, A. Buggenhout, D. Franchimont, J. C. Nöel

**Affiliations:** 1 Department of Gynecology, Academic Hospital Erasme, Université Libre de Bruxelles (ULB), 808, Route de Lennik, 1070 Brussels, Belgium; 2 Department of Digestive Surgery, Academic Hospital Erasme, Université Libre de Bruxelles (ULB), 808, Route de Lennik, 1070 Brussels, Belgium; 3 Department of Gastroenterology, Academic Hospital Erasme, Université Libre de Bruxelles (ULB), 808, Route de Lennik, 1070 Brussels, Belgium; 4 Department of Pathology, Academic Hospital Erasme, Université Libre de Bruxelles (ULB), 808, Route de Lennik, 1070 Brussels, Belgium

**Keywords:** Deep infiltrating endometriosis, Stomach, Cyclical pain

## Abstract

Gastric endometriosis is a very rare event. It enters in the differential diagnosis of cyclical or chronic epigastric pain, especially in the context of endometriotic patients. The diagnosis of a gastric submucosal mass requires further investigations to rule out the presence of malignancy or associated adenocarcinoma. Because of it can be associated with transverse colon endometriosis and/or diaphragmatic endometriosis, careful examination of the upper abdomen at laparoscopy should be emphasized. We report here a very rare case of gastric endometriosis associated with transverse colon endometriosis.

## Introduction

**Fig. 1:**
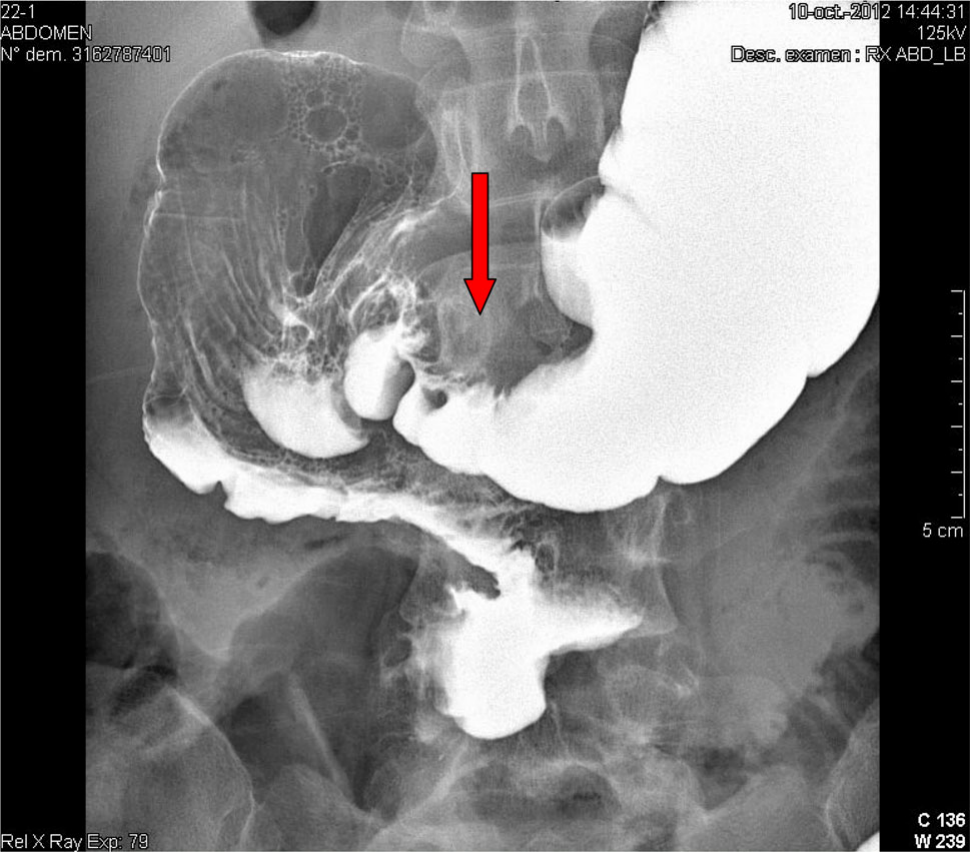
Double-contrast barium enema showing deep endometriotic involvement of the transverse colon (*red arrow*)

Endometriosis is defined as the presence of endometrial-like glands and stroma outside the uterus. Deep infiltrating endometriosis (DIE) is defined as the presence of endometriosis more than 5 mm under the peritoneum. DIE can be a cofactor for infertility, but is mainly responsible for pain and very often severe pain [1–3]. The most frequent localizations of endometriosis are the peritoneum and the ovaries. Extragenital lesions are not rare and the digestive tract is the most frequently affected. The rectosigmoïd junction and the rectum are the first localizations in terms of frequency, followed by the sigmoid and the right colon including the caecum and the appendix [4]. On the other hand, the transverse colon and the stomach are rarely affected. Clinical examination is essential to detect nodules or indurations that could be suggestive of DIE. The preoperative setup of DIE often includes pelvic MRI and endovaginal ultrasound or transrectal ultrasonography. In case of DIE, the preoperative setup is generally focused on the affected organ. Concerning the large bowel, colonoscopy is often negative because of the lesion comes from the inside of the abdominal cavity while rectal or colonic malignancies originate from the colonic mucosa and can be quite easily visible and biopsied by colonoscopy [5]. Double-contrast barium enema (DCBE), can detect the presence of multiple lesions (even on the ileocaecal region), it can be able to measure the length and the importance of the narrowing of the large bowel lumen after insufflation, as well as the distance between the bottom of the lesion and the anal verge. Positive barium enema is always associated with an infiltration of the large bowel wall [5]. In this case, barium enema showed the presence of a huge and long large bowel stenosis on the transverse colon (Fig. 1). Gastric endometriosis is a very rare lesion affecting just some women. It can be responsible for perimenstrual nausea and blood vomiting. It is generally a peroperative discovery and there are very few data in the literature on gastric endometriosis [6]. We describe a case of a young woman with multiple colonic endometriotic stenoses and in particular with a huge lesion involving the transverse colon (Fig. 1), the gastrocolic ligament as well as the stomach. This is the first reported case of transverse colon endometriosis associated with gastric endometriosis and the second reported case of gastric endometriosis.

## Patient and methods

**Fig. 2:**
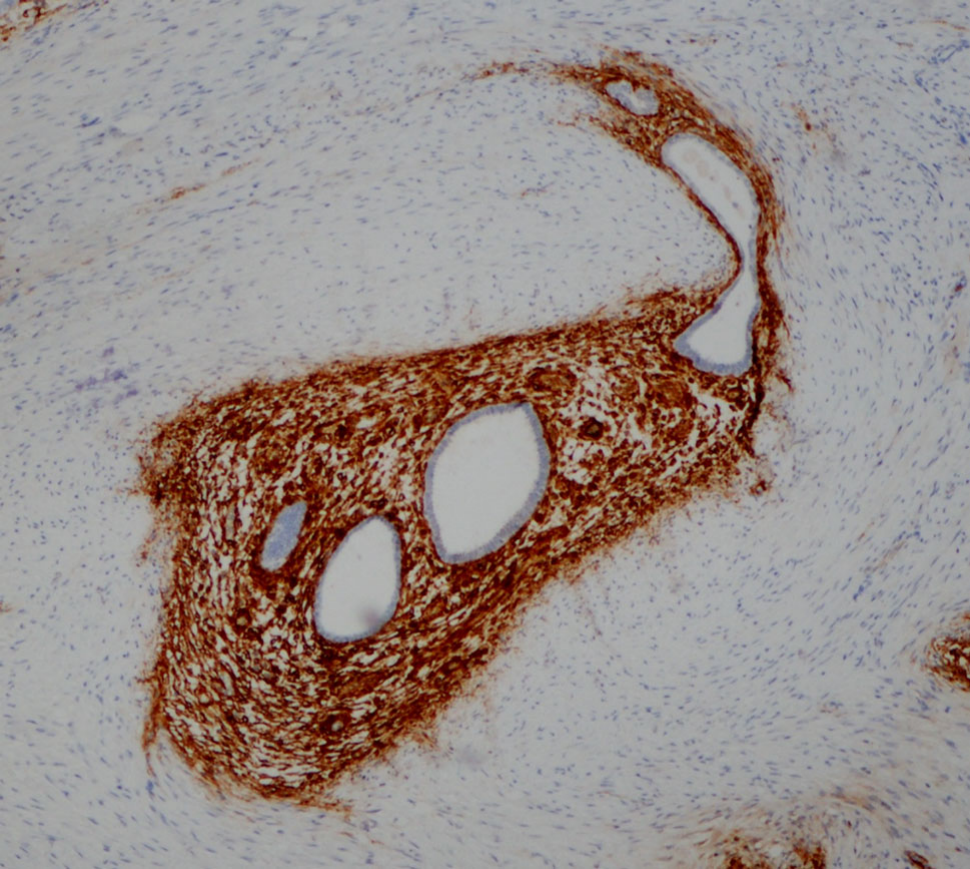
CD-10 immunohistochemistry staining the endometriotic stromal cells of endometriosis infiltration of the gastric muscularis

A 28-year-old patient consulted the gastroenterologist for a 4-year history of general abdominal pain, constipation, pain at defecation, alternation of constipation and diarrhea, dyspepsia and epigastric postprandial pain. On examination, the abdomen was tender, but with no signs of peritonitis. Pain increased during menses which orientated the patient to the gynecologist. There was a presence of a 4-cm diameter bluish induration in the posterior vaginal fornix which was exquisitely painful at palpation. Colonoscopy was impossible because of the presence of a severe narrowing of the rectosigmoïd. DCBE showed the presence of an important stenosis at the levels of the rectosigmoïd and the transverse colon (Fig. 1). Pelvic MRI showed the same findings in the pelvis, but did not examine the upper abdomen. Laparoscopy showed the presence of a fixed pelvis with a severe rectosigmoïd stenosis, a deep infiltrating mid transverse colon stenosis of 6 cm length (Fig. 1), an infiltration of the gastrocolic ligament and an induration of the stomach antrum over a length of about 6 cm with serosal lesions suggestive of endometriosis. Conversion to laparotomy was decided. Rectosigmoïd resection with end-to-end mechanical anastomosis, segmental resection of the transverse colon with manual end-to-end suture and partial gastrectomy were performed along with a gastrocolic ligament ablation. Histology and CD-10 immunohistochemistry revealed the presence of deep infiltrating endometriosis in all resected specimen (Fig. 2). In the stomach, all layers were extensively involved by endometriosis except the mucosal layer. The patient rapidly improved after surgery and is still symptoms free after 1-year follow-up.

## Discussion and conclusions

Endometriosis of the stomach is a very uncommon event. The digestive tract represents the third location of endometriosis after the peritoneum and the ovaries. The large bowel is from far the most frequently extragenital affected organ with a predilection for the rectosigmoïd and sigmoid. To our knowledge, this is the first case of gastric endometriosis with an involvement of the transverse colon, and the second case of gastric endometriosis described in the available literature [7]. Gastric endometriosis is probably underrated because gynecologists are mostly focused on the pelvis and all the aspects of the stomach are not systematically checked at laparoscopy. It is of course difficult to determine in the present case which organ invaded the other one since the sizes of both lesions were identical at macroscopical examination. As endometriosis very rarely involves the mucosa [5], it can be considered as a submucosal lesion. Until now, no case of gastric mucosal involvement by endometriosis has been described in the available literature. In the presence of a submucosal gastric mass, a differential diagnosis must be done with gastric adenomyoma, hamartomata, leiomyoma, heterotopic pancreatic tissue and GIST (gastrointestinal stromal tumor) [8–11]. In this case, the diagnosis of endometriosis was based on the micro- and macroscopic aspects of the lesion, the clinical presentation, the general context of endometriosis and on CD-10 immunohistopositivity which is the most reliable marker for endometriotic stromal cells [12, 13]. All other submucosal lesions are intramural lesions, while gastric DIE involves the gastric peritoneum and can thus be suspected at laparoscopy. All these lesions can present with intermittent epigastric pain, heart burn with occasional vomiting [8–11]. Another differential diagnosis is diaphragmatic endometriosis which can also be responsible for cyclical epigastric and/or shoulder pain which emphasizes the importance of upper abdomen examination at laparoscopy [14]. What seems to be specific of gastric endometriosis is the cyclical character of gastric symptoms as well as the presence of other endometriotic lesions. May be the most difficult differential diagnosis is gastric adenomyomata, also a rare lesion that can affect the gastric antrum or the pylorus. As gastric endometriosis, gastric adenomyomata histologically contains smooth muscles and glandular tissue [15], but also ductal tissue as well as pancreatic acini and/or Brünner’s glands. If there is a predominance of pancreatic tissue then the term of “pancreatic heterotopia” is more appropriate [15]. The histogenesis of gastric adenomyomata is unknown, some authors proposed that they should be regarded as hamartomata [9], while others favored heterotopia [10]. Most reports consider them to be of developmental origin [11]. It is of course difficult to draw definitive conclusions based on a reported case. However, according to the available literature published on this topic, it appears that the diagnosis of a gastric lesion should require further investigations including biopsies to rule out malignancy, and very often a surgical excision of the lesion because some of these lesions can be associated with adenocarcinoma [16]. Clinicians dealing with DIE should be aware of the possibility of stomach endometriosis in a patient presenting with cyclical gastric symptoms in a general context of endometriosis.
